# Supplementary material for: Causal interplay between lactose intolerance and gut microbiota: a combined bidirectional Mendelian randomization and in vivo validation study
Source: Front Nutr. 2026 Jun 1;13:1803337. doi: 10.3389/fnut.2026.1803337 (PMC13265576; doi:10.3389/fnut.2026.1803337)
Supplement: Supplementary file 1 [file Data_Sheet_1.zip › supplementary materials/Forward/leave one out cross validation/ebi-a-GCST90027563.finngen_R12_E4_LACTONAS.pdf]

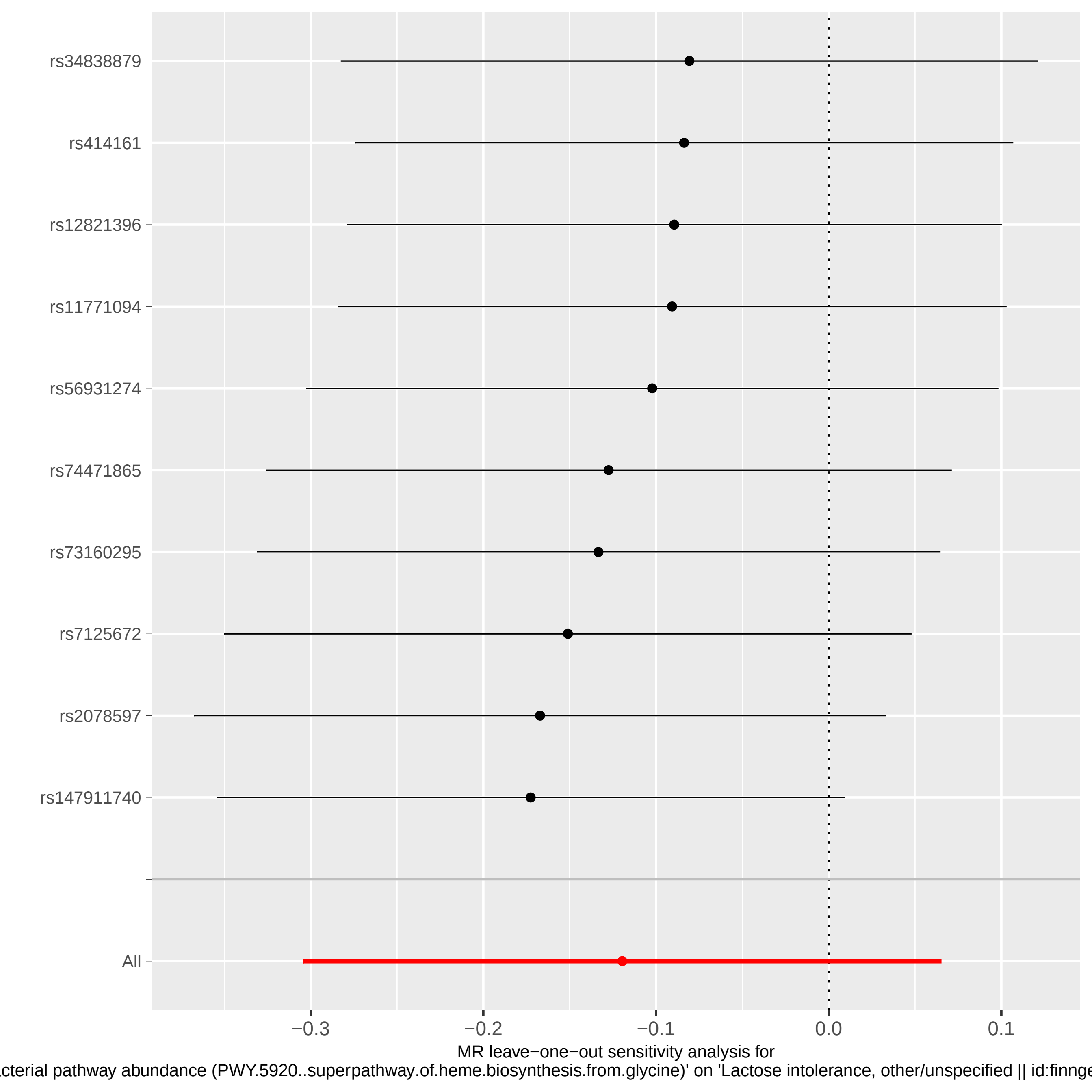
rs34838879
rs414161
rs12821396
rs11771094
rs56931274
rs74471865
rs73160295
rs7125672
rs2078597
rs147911740
All
−0.3
−0.2
−0.1
0.0
0.1
MR leave−one−out sensitivity analysis for
cterial pathway abundance (PWY.5920..superpathway.of.heme.biosynthesis.from.glycine)' on 'Lactose intolerance, other/unspecified || id:finnge
